# Interpretable factor models of single-cell RNA-seq via variational autoencoders

**DOI:** 10.1093/bioinformatics/btaa169

**Published:** 2020-03-16

**Authors:** Valentine Svensson, Adam Gayoso, Nir Yosef, Lior Pachter

**Affiliations:** b1 Division of Biology and Biological Engineering, California Institute of Technology, Pasadena, CA 91125, USA; b2 Center for Computational Biology; b3 Department of Electrical Engineering and Computer Sciences, University of California, Berkeley, CA 91125, USA; b4 Chan Zuckerberg Biohub, San Francisco, CA 94158, USA; b5 Department of Computing and Mathematical Sciences, California Institute of Technology, Pasadena, CA 91125, USA

## Abstract

**Motivation:**

Single-cell RNA-seq makes possible the investigation of variability in gene expression among cells, and dependence of variation on cell type. Statistical inference methods for such analyses must be scalable, and ideally interpretable.

**Results:**

We present an approach based on a modification of a recently published highly scalable variational autoencoder framework that provides interpretability without sacrificing much accuracy. We demonstrate that our approach enables identification of gene programs in massive datasets. Our strategy, namely the learning of factor models with the auto-encoding variational Bayes framework, is not domain specific and may be useful for other applications.

**Availability and implementation:**

The factor model is available in the scVI package hosted at https://github.com/YosefLab/scVI/.

**Contact:**

v@nxn.se

**Supplementary information:**

Supplementary data are available at *Bioinformatics* online.

## 1 Introduction

The study of the regulatory architecture of cells has revealed numerous examples of co-regulation of transcription of large numbers of genes (Jang *et al.*, 2017; Kondo *et al.*, 2018), and this has been used to link the organization of cells to their distinct functions in response to developmental or external stimuli (Romero *et al.*, 2012). While studies of cells in bulk have led to interesting population-level insights about the relationships between genes (Thompson *et al.*, 2015), the study of individual cells via single-cell RNA-seq has led to questions about the dependence of relationships between genes on cell type (Lindgren *et al.*, 2017).

Principal component analysis (PCA) is a popular linear method for dimensionality reduction in single-cell RNA-seq (Andrews and Hemberg, 2017; Rostom *et al.*, 2017). As a result of its efficiency, PCA has been used for exploratory data analysis to visualize the structure of high-dimensional data in two or three dimensions. PCA also provides a linear model of the data; a key feature of the method that can be used for prediction (Tipping and Bishop, 1999). In the case of single-cell RNA-seq, datapoints correspond to cells and the coordinates of each cell represent the gene expression levels for each gene in the transcriptome. Thus, PCA can be used to study structured variation between cells by revealing differences along axes of greatest variation. In PCA, linear weight parameters (loadings) are used to predict gene expression in each cell, conditional on the latent variables (coordinates) per cell. The loadings corresponding to the principal component axes can be interpreted as ‘meta-genes’: sets of genes which tend to be expressed together (Brunet *et al.*, 2004; Raychaudhuri *et al.*, 2000). Thus, PCA of gene expression provides a formal mathematical framework for studying the biological idea of ‘gene programs’ (Stuart *et al.*, 2003) by simultaneously explaining structured variation between cells and genes (Guo *et al.*, 2010; Islam *et al.*, 2011).

While PCA is easy to use and is often applied to single-cell RNA-seq data, the method has some drawbacks. PCA models data as arising from a continuous multivariate Gaussian distribution, and thus optimizes a Gaussian likelihood (Pearson, 1901; Tipping and Bishop, 1999). This model assumption is at odds with the count data measured in single-cell RNA-seq (Svensson, 2020; William Townes *et al.*, 2019), and leads to interpretation problems (Hicks *et al.*, 2018). To address this issue, a number of methods define factor methods tailored to single-cell transcriptomics data (Buettner *et al.*, 2017; Durif *et al.*, 2019; Pierson and Yau, 2015; Zhu *et al.*, 2017). For example, ZINB-WaVE defines a linear factor model where gene weights are parameters, cell factor values are latent variables and data arise from a zero-inflated negative binomial distribution (Risso *et al.*, 2018). However, as single-cell transcriptomics datasets have grown in size to hundreds of thousands of observations (Svensson *et al.*, 2018), efficiency and scalability considerations have become paramount and inference with parametric models can be intractable. To address scalability requirements, new methods based on variational autoencoders have been developed; these leverage the large amounts of available data to learn non-linear maps, and crucially scale well thanks to efficient algorithms for inference that leverage the structure of autoencoders (Eraslan *et al.*, 2019; Lopez *et al.*, 2018).

Autoencoders consist of a pair of functions: a representation function and a reconstruction function, which are typically parameterized as neural networks (Hinton and Zemel, 1993). The two autoencoder functions can be seen as a non-linear generalization of the two projections associated with PCA (Plaut, 2018). By optimizing the pair of neural networks, efficient low-dimensional representations of data can be identified. A variational autoencoder (VAE) uses a similar strategy but with latent variable models (Kingma and Welling, 2013). Each datapoint is represented by a set of latent variables which can be decoded by neural networks to produce parameters for a probability distribution, thus defining a generative model. To infer the latent variable values (the representation), a neural network is used to find per-datapoint parameters for a probability distribution in the representation space. This defines an ‘inference model’ which attempts to approximate the posterior distribution of the latent variables given the observed data with a variational distribution (Marino *et al.*, 2018).

**Fig 1.**
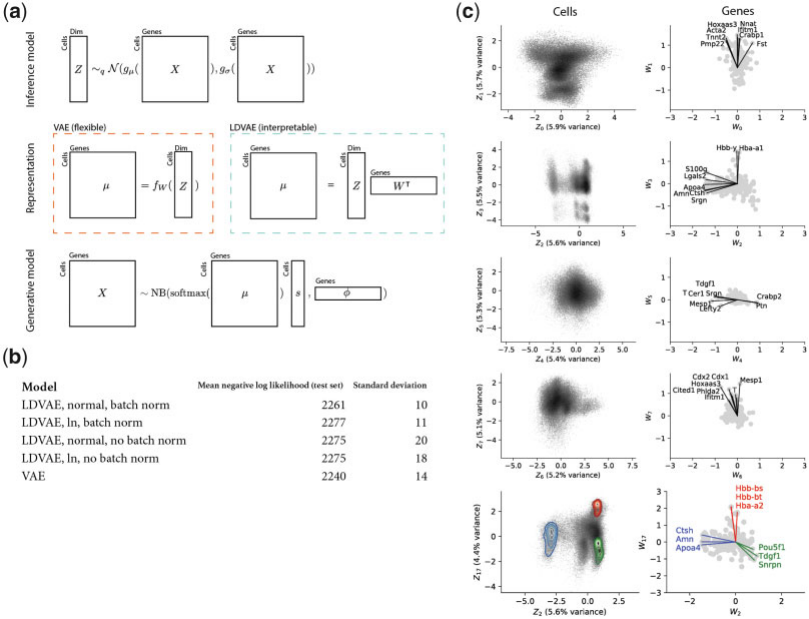
(**a**) A sketch of the general architecture of scVI autoencoders with two alternative representation models. (**b**) Comparison of reconstruction error on the Pijuan-Sala *et al.* (2019) data with VAE and the four variants of LDVAE after running 100 epochs. (**c**) Results from fitting a 20-dimensional LDVAE. (Left column): Density plots of the cells in representation space. (Right column): Scatter plots of gene loadings corresponding to the representation coordinates. The last row shows a pair of factors which discriminates three cell types annotated by Pijuan-Sala *et al.* (2019) (red: erythroid, blue: extraembryonic endoderm, green: epiblast). Top genes indicated as vectors with names. (Color version of this figure is available at *Bioinformatics* online.)

Inference using VAEs scales to arbitrarily large data since mini-batches of data can be used to train the parameters for both the inference model and the decoder function (Kingma and Welling, 2013). However despite these efficiency advantages, the representations inferred with VAEs are not directly interpretable. While efforts have been made to develop interpretable VAE’s (Ainsworth *et al.*, 2018), the difficulty in interpreting VAE representations continues to be a major drawback of VAE’s. We show that using a flexible non-linear inference model along with a linear reconstruction function makes it possible to benefit from the efficiency of VAEs, while retaining the interpretability provided by factor models. Specifically, by adapting the method of scVI (Lopez *et al.*, 2018), we demonstrate a scalable approach to learning a latent representation of single-cell RNA-seq data, that identifies the relationship between cell representation coordinates and gene weights via a factor model. Our approach results in a tradeoff: whereas typically autoencoder models are designed with the same network topology in the inference functions and the reconstruction functions, what we propose is a restricted reconstruction function that leads to an increase in reconstruction error. However, by virtue of being linear, our reconstruction function provides an interpretable link between gene programs and cellular molecular phenotypes (Fig. 1a).

## 2 Materials and methods

The generative model of scVI, when data are from a single batch and zero-inflation is deactivated, is

$$\begin{array}{c} z_{n}∼\text{Normal}\left(0,I\right),\\ s_{n}∼\;\text{log}\;\;\text{normal}\left(s_{\mu },s_{\sigma }^{2}\right),\\ \mu_{n}=\text{softmax}\left(f_{W}\left(z_{n}\right)\right),\\ v_{n}^{g}∼\text{Gamma}\left(\theta^{g},\mu_{n}^{g}\right),\\ y_{n}^{g}∼\text{Poisson}\left(v_{n}^{g}\cdot s_{n}\right). \end{array}$$

In this model, $s_{n}$ is a random variable for the exposure or count depth of a cell, with priors $s_{\mu }$ and $s_{\sigma }$. The random variable $z_{n}$ provides a D-dimensional representation of cells. The parameter $\theta^{g}$ represents the overdispersion of a gene, and the Gamma is parameterized by its shape and mean. We replace the neural network $f_{W}\left(z_{n}\right)$ with a linear function:

$$\mu_{n}=z_{n}W^{T}.$$

This way the expression level $\mu_{n}^{g}$ of a gene g in a cell n is affected by the weights $w_{g}^{d}$ depending on the coordinate $z_{n}^{d}$ of a cell n, giving a direct link between cell representation and gene expression.

We also considered a variation of this model where the latent variables $z_{n}$ are distributed as a logistic normal (ln) distribution. In this case, each $z_{n}$ has positive values and sums to 1, making it similar to semi-non-negative matrix factorization (Levitin *et al.*, 2019; Srivastava and Sutton, 2017). Such a model adds a further layer of interpretability; the cells are embedded in a simplex, where the nodes of the simplex represent archetypal cell types (Korem *et al.*, 2015). In addition, we investigated the effect of applying a batch-norm transformation of the linearly decoded parameters (Ioffe and Szegedy, 2015).

## 3 Results

To explore the potential for interpretability in the VAE framework, we implemented a linearly decoded variational autoencoder (LDVAE) in scVI. The model was applied to two datasets of single-cell RNA-sequencing from a large number of developing mouse embryos in different stages of development (Cao *et al.*, 2019; Pijuan-Sala *et al.*, 2019). The first dataset (Pijuan-Sala *et al.*, 2019) consists of 125 775 cells from 411 mouse embryos undergoing gastrulation measured using the commercial 10× Genomics platform and sequenced relatively deeply (11% non-zero values).

A comparison of the VAE with the LDVAE methods showed that VAE indeed has a smaller reconstruction error than the LDVAE methods (Fig. 1b). Among the LDVAE method variants, using a normal latent distribution and batch norm has the smaller reconstruction error (on held-out data). Between the LDVAE models with ln distributed latent space, the comparison was inconclusive for the Pijuan-Sala *et al.* (2019) data, but batch norm performed better for the Cao *et al.* (2019) data (Supplementary Fig. S1). With either VAE or LDVAE, the representation Z can be used to learn which cells are similar to each other and can be used for clustering. For example, erythroid cells, extraembryonic endoderm cells and epiblast cells annotated by the original authors can be separated by factors 2 and 17. However, the axes of representation learned by the LDVAE model can be directly related to axes of co-expressed genes (Fig. 1c). For example, variation along the $Z_{2}$ axis is related to simultaneous variation in expression of Pou5f1 and Tdgf1, two genes important for epiblast development (Bianco *et al.*, 2002; Wu and Schöler, 2014). Variation along the $Z_{17}$ axis is related to co-variation in beta globin (Hbb) genes which are key components of erythroid cells. Additionally, variation between epiblast and erythroid cells along $Z_{2}$ is orthogonal to variation between epiblast and extraembryonic endoderm cells along $Z_{17}$, two independent lineages in embryonic development.

While the ln latent distribution results in higher reconstruction error than the normal distribution, it has benefits for interpretation. Since a factor z is restricted to non-negative values, genes with negative weights w can only decrease in expression as a function of z. This way cells using a particular regulatory program can more effectively be grouped to specific factors (Supplementary Fig. S2). We also found that using batch-norm transformation improved model performance.

The learned Z representations from the different models can be compared by investigating the covariance matrix ${\hat{Z}}^{T}\hat{Z}$ (where $\hat{Z}$ is a centered and scaled version of Z). This illustrates that LDVAE learns representations with fewer covarying factors $z_{d}$ (Supplementary Fig. S3). Unlike linear methods, the VAE is not constrained by covarying factors since the non-linear neural network $f_{W}\left(\cdot \right)$ can produce vastly different gene expressions along a linear path in the Z representation. Comparing the proposed alternative LDVAE models, using a normal latent distribution induces less correlation between factors. By performing eigen decomposition on a covariance matrix the proportion of variance explained by each factor can be quantified. This allows ordering of factors which can be used to identify the regulatory programs with the most variation across the dataset. It also illustrates the simplicial structure of ln distributed latent variables since one factor is always linearly dependent on the other factors (Supplementary Fig. S3).

The second dataset (Cao *et al.*, 2019) consists of 1 949 131 cells from 61 embryos in total using the sci-RNA-seq method at shallow sequencing (2% non-zero values). This dataset is, to our knowledge, the largest scRNA-seq study published to date. To illustrate the scalability of our model, we fit a 10-dimensional LDVAE to the data which allows identification of cells similar to each other and for the determination of covarying genes (Supplementary Fig. S4).

Cells were also subsampled to different numbers before fitting LDVAE models. We found that inference runs in linear time, with 5 s per 1000 cells to reach 10 epochs using a CPU (Intel Core i7-7800X). Using a consumer-grade GPU (NVIDIA GeForce RTX 2070), inference only requires 2 s per 1000 cells to reach 10 epochs, with a total time of less than an hour for the full dataset. The inference times did not depend on the sparsity of the data, as the neural architecture is fixed, and operations are invariant to observed values (Supplementary Fig. 5a). Investigating the reconstruction error curves per epoch, the models converged after 2–3 epochs for datasets larger than 100 000 cells (Supplementary Fig. 5b). Determining a minimal number of epochs is a difficult general problem, but our results suggest a rule of thumb of ‘1 million divided by the number of cells in the dataset’ epochs for first pass analysis.

Jupyter notebooks to produce the results are available at https://github.com/pachterlab/SGYP_2019 as well as Figshare at https://doi.org/10.6084/m9.figshare.11725920.v1. For convenience, the embryo data from Pijuan-Sala *et al.* (2019) and Cao *et al.* (2019) are also available in an H5AD object on the Figshare accession and on Google Cloud Storage at gs://h5ad/2019-02-Pijuan-Sala-et-al-Nature/pijuan_sala_atlas.h5ad and gs://h5ad/2019-02-Cao-et-al-Nature/cao_atlas.h5ad. A general tutorial on how to use the LDVAE model is available in the scVI Github repository at https://github.com/YosefLab/scVI/blob/master/tests/notebooks/linear_decoder.ipynb.

## 4 Discussion

Our results show that interpretable non-Gaussian factor models can be linked to variational autoencoders to enable interpretable, efficient and multivariate analysis of large datasets. This is useful for the investigation of gene co-expression in large scRNA-seq datasets, and the approach we have outlined should be applicable in other settings where interpretability is paramount.

## Supplementary Material

btaa169_Supplementary_Data
