# Supplementary figures and images for: Interpretable factor models of single-cell RNA-seq via variational autoencoders
Source: Bioinformatics. 2020 Mar 16;36(11):3418–21. doi: 10.1093/bioinformatics/btaa169 (PMC7267837; doi:10.1093/bioinformatics/btaa169)

(a)

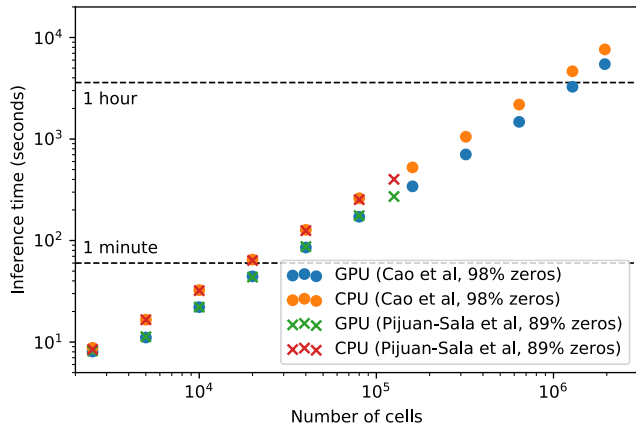

(b)

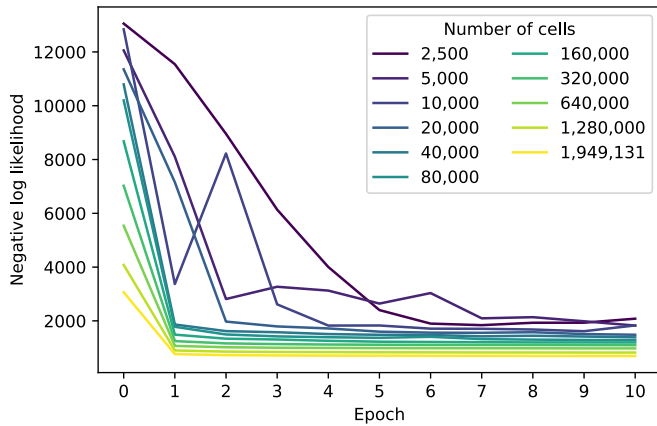

Supplement: btaa169_Supplementary_Data [file btaa169_supplementary_data.zip › btaa169-Suppl_Data/Supp. Fig 5.pdf]

(a)

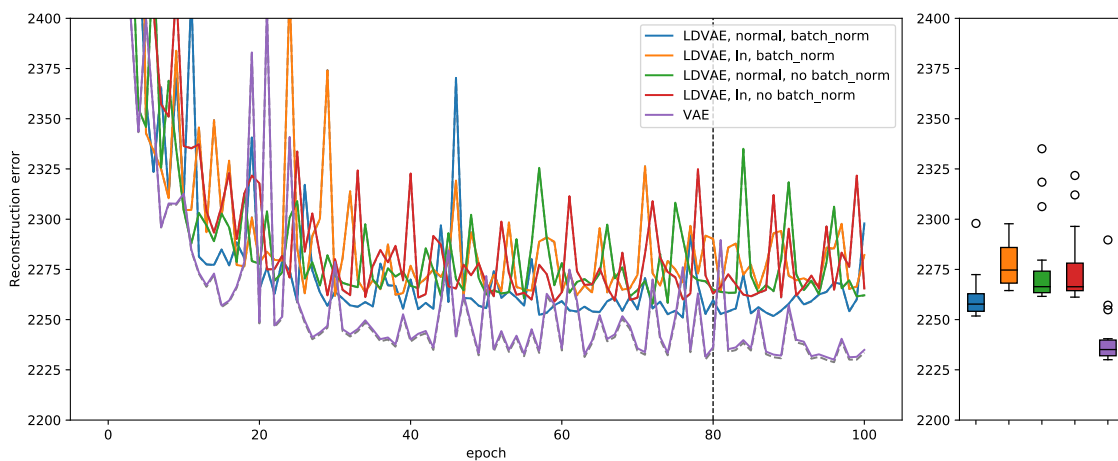

(b)

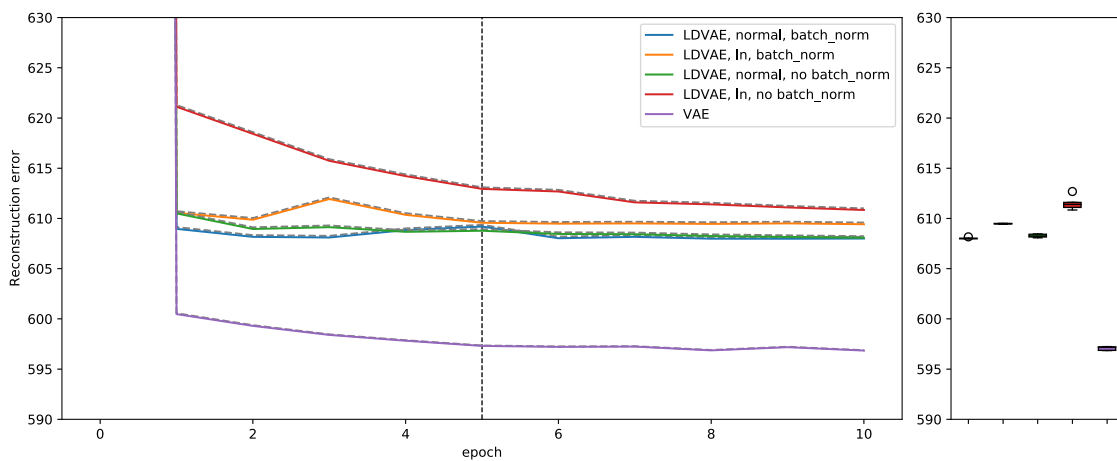

Supplement: btaa169_Supplementary_Data [file btaa169_supplementary_data.zip › btaa169-Suppl_Data/Supp. Fig 1.pdf]

## Cells

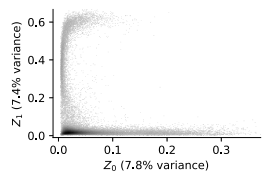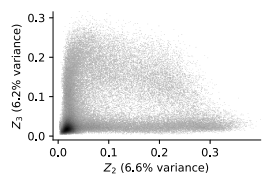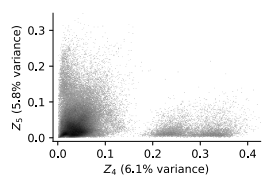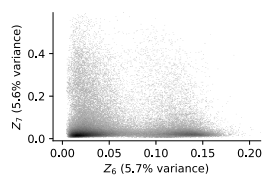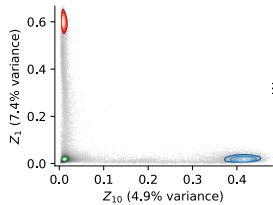

## Genes

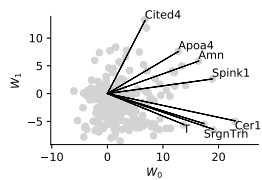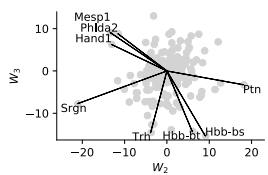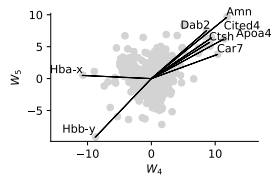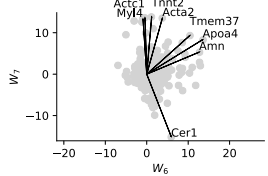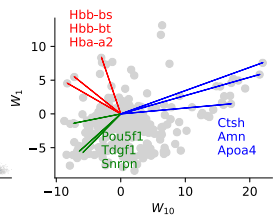

Supplement: btaa169_Supplementary_Data [file btaa169_supplementary_data.zip › btaa169-Suppl_Data/Supp. Fig 2.pdf]

(a) Pijuan-sala *et al* 2019

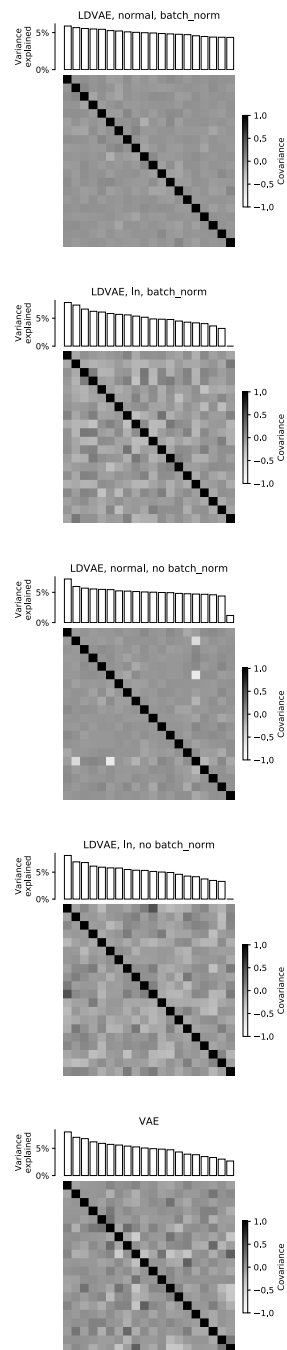

(b) Cao *et al* 2019

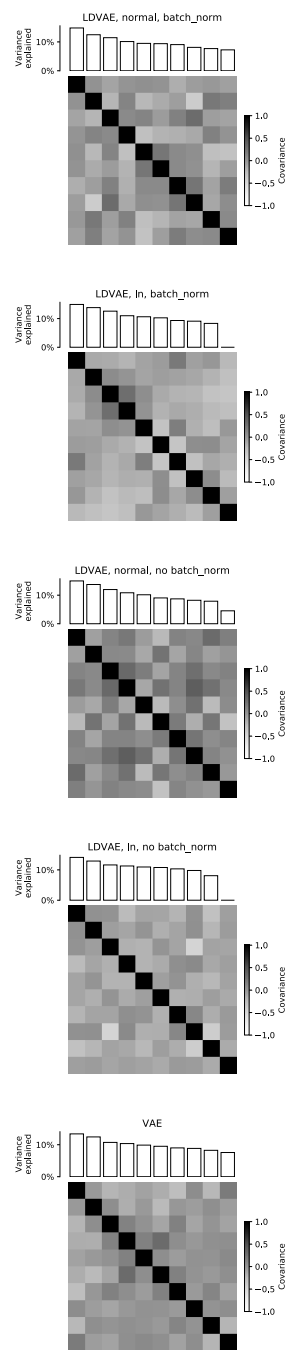

Supplement: btaa169_Supplementary_Data [file btaa169_supplementary_data.zip › btaa169-Suppl_Data/Supp. Fig 3.pdf]

(a)

Cao *et al* 2019 LDVAE (normal)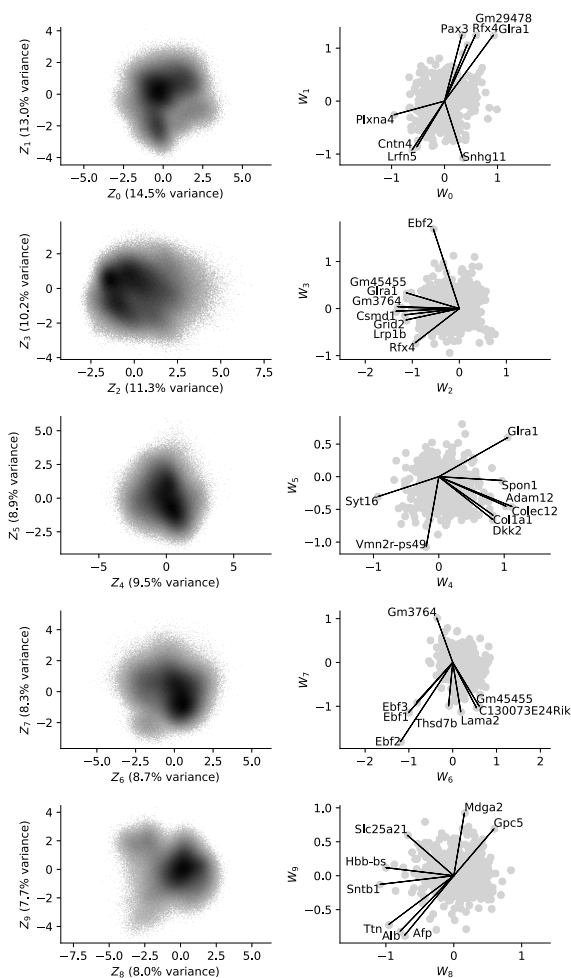

(b)

Cao *et al* 2019 LDVAE (ln)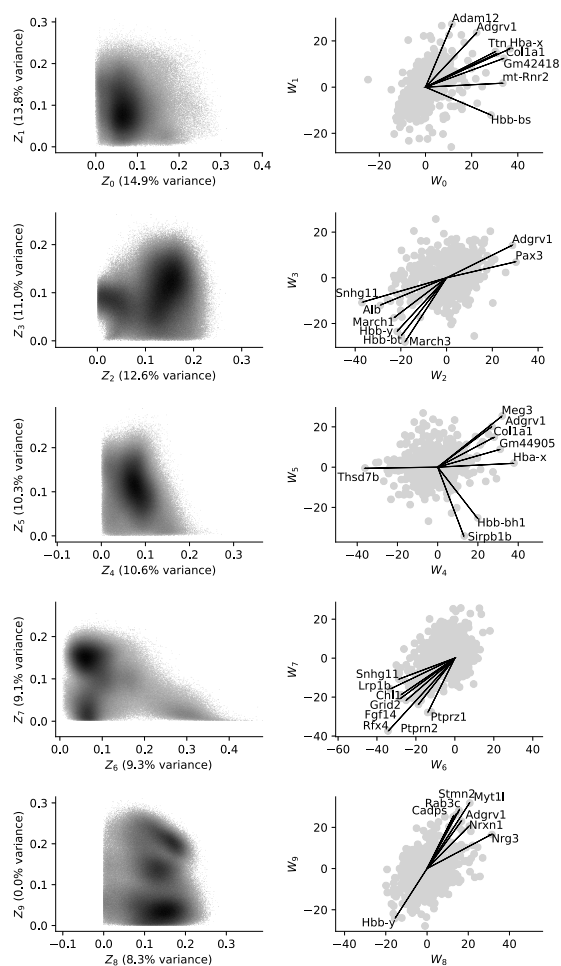

Supplement: btaa169_Supplementary_Data [file btaa169_supplementary_data.zip › btaa169-Suppl_Data/Supp. Fig 4.pdf]
